# Associations Between Maternal Depressive Symptoms and Nonresponsive Feeding Styles and Practices in Mothers of Young Children: A Systematic Review

**DOI:** 10.2196/publichealth.6492

**Published:** 2017-05-26

**Authors:** Ana Cristina Lindsay, Tatiana Mesa, Mary L Greaney, Sherrie F Wallington, Julie A Wright

**Affiliations:** ^1^ Exercise and Health Sciences College of Nursing and Health Sciences University of Massachusetts Boston Boston, MA United States; ^2^ Department of Kinesiology Kinesiology/Health Studies University of Rhode Island Kingston, RI United States; ^3^ Lombardi Comprehensive Cancer Center Georgetown University Medical Center Georgetown University Washington, DC United States

**Keywords:** maternal depression, child, feeding behavior, practices, feeding styles, obesity

## Abstract

**Background:**

Childhood obesity is a significant global public health problem due to increasing rates worldwide. Growing evidence suggests that nonresponsive parental feeding styles and practices are important influences on children’s eating behaviors and weight status, especially during early childhood. Therefore, understanding parental factors that may influence nonresponsive parental feeding styles and practices is significant for the development of interventions to prevent childhood obesity.

**Objective:**

The objectives of this systematic review were to (1) identify and review existing research examining the associations between maternal depressive symptoms and use of nonresponsive feeding styles and practices among mothers of young children (2-8 years of age), (2) highlight the limitations of reviewed studies, and (3) generate suggestions for future research.

**Methods:**

Using the PRISMA (Preferred Reporting Items for Systematic review and Meta-Analysis Protocols) guidelines, six electronic academic databases were searched for peer-reviewed, full-text papers published in English between January 2000 and June 2016. Only studies with mothers 18+ years old of normally developing children between 2 and 8 years of age were included. Of the 297 citations identified, 35 full-text papers were retrieved and 8 were reviewed.

**Results:**

The reviewed studies provided mixed evidence for associations between maternal depressive symptoms and nonresponsive feeding styles and practices. Two out of three studies reported positive associations with nonresponsive feeding styles, in that mothers with elevated depressive symptoms were more likely than mothers without those symptoms to exhibit uninvolved and permissive or indulgent feeding styles. Furthermore, results of reviewed studies provide good evidence for association between maternal depressive symptoms and instrumental feeding (3 of 3 reviewed studies) and nonresponsive family mealtime practices (3/3), but mixed evidence for pressuring children to eat (3/6) and emotional feeding (1/3). In addition, evidence for the association between maternal depressive symptoms and restricting child food intake was mixed: one study (1/6) found a positive association; two studies (2/6) found a negative association; whereas one study (1/6) found no association.

**Conclusions:**

This review indicates that the results of studies examining the associations between maternal depressive symptoms and parental feeding styles and practices are mixed. Limitations of studies included in this review should be noted: (1) the use of a diverse set of self-report questionnaires to assess parental feeding practices is problematic due to potential misclassification and makes it difficult to compare these outcomes across studies, thus caution must be taken in drawing conclusions; and (2) the majority of included studies (6/8) were cross-sectional. There is a need for additional longitudinal studies to disentangle the influence of depression on parental feeding styles and practices. Nevertheless, given that depressive symptoms and feeding styles and practices are potentially modifiable, it is important to understand their relationship to inform obesity prevention interventions and programs.

## Introduction

Childhood obesity is an important global public health issue due to existing prevalence and increasing rates worldwide [1,2]. The increasing prevalence of childhood obesity in young children is particularly concerning, given the evidence that children’s weight status is associated with weight status in adulthood, making early childhood a critical period for prevention of overweight and obesity [2-4]. Consequently, identifying modifiable factors associated with increased risk of early childhood obesity is a priority [1-4].

Early childhood is an important period of growth and development that influences one’s health during childhood and beyond [3-7]. It is when the foundations for healthful eating habits that have long-lasting implications for weight status and related comorbidities are established [4,5,7-9]. Several parental characteristics are associated with children’s risk of overweight and obesity including parents’ weight status [1-4], sociodemographic and economic characteristics (eg, income, education) [1-4], and mental health status (eg, depression) [10-19]. Parents, especially mothers, influence their children’s development and maintenance of eating habits and food preferences [2,4-9,10]. Parental feeding styles and parental feeding practices have been identified as particularly important influences on children’s eating behaviors during early childhood [5,7,8,9-16,20-24].

Parental feeding style, the overarching feeding strategy parents adopt during feeding situations [9,11,20,21], has been conceptualized as having two main dimensions: demandingness (also defined as control) and responsiveness (also defined as warmth). Within these two dimensions, there are four parental feeding styles typologies: (1) authoritative (high level of demandingness and high level of responsiveness), (2) authoritarian (high level of demandingness and low level of responsiveness), (3) indulgent or permissive (low demandingness and high responsiveness), and (4) uninvolved or neglectful (low demandingness and low responsiveness). Parental feeding practices are specific behaviors that parents use to influence the amount and/or type of food a child eats and include monitoring and controlling food intake, pressuring to eat, instrumental and emotional feeding, and so on [9,11,21-25].

Family meals and family mealtime practices are key family routines relevant to obesity prevention [26]. Family mealtime practices encompass habits and processes that a family engages in around eating together [27]. Family mealtime may offer several benefits to children’s health and development such as helping children develop healthful eating patterns and weight status [27-29].

Understanding factors that may be associated with parental feeding styles and practices, and family mealtime practices that are unintentionally detrimental to children’s development of healthful eating habits is of great importance to the development of interventions to prevent child obesity. Providing parents with guidance on healthful feeding styles and practices will help children develop healthful eating habits and, ultimately, maintain a healthy weight status [2-4,8,30,31].

Research suggests that mental health status of the parents may influence the weight status of their child through parental feeding styles and practices [7,10-13]. Mental health conditions (eg, depression and depressive symptoms) among mothers of young children are increasingly recognized as an important public health concern [32-36]. According to the National Institute of Mental Health one in seven women of reproductive age are affected by depression, and 15% of women in the United States experience postpartum depression [32,36]. Depressive symptoms can affect mothers’ sensitivity and responsiveness to their children and can contribute to less engaged or responsive mother-child interactions as well as a higher use of disengaged (eg, uninvolved and permissive/indulgent) or controlling (eg, authoritarian) parenting behaviors [10-14]. In addition, elevated depressive symptoms such as low energy and diminished pleasure in activities may contribute to decreased maternal-child involvement [10-13], with mothers choosing strategies for coping that require less cognitive effort [11-13].

Given the high prevalence (15%-38%) of depression and depressive symptoms among women of childbearing age [32-36] and increasing evidence linking maternal depressive symptoms to nonresponsive feeding styles and practices related to the risk of childhood obesity [11-14,30,37], the objectives of this systematic literature review were to (1) identify and review existing research examining the associations between maternal depressive symptoms and use of nonresponsive feeding styles and practices among mothers of young children (2–8 years of age), (2) highlight the limitations of reviewed studies, and (3) generate suggestions for future research.

## Methods

### Study Design

We conducted this review by following the reporting guidelines of the Preferred Reporting Items for Systematic Reviews and Meta-Analysis (PRISMA) statement [38]. These guidelines include a four-phase flow diagram to guide the inclusion and exclusion of research papers [38]. In addition, the guidelines provide a 27-item checklist outlining standards per review section (eg, title, abstract, introduction, methods, results, discussions, funding) to ensure that reviews are systematically conducted and reported [38].

### Search Strategy

**Figure 1:**
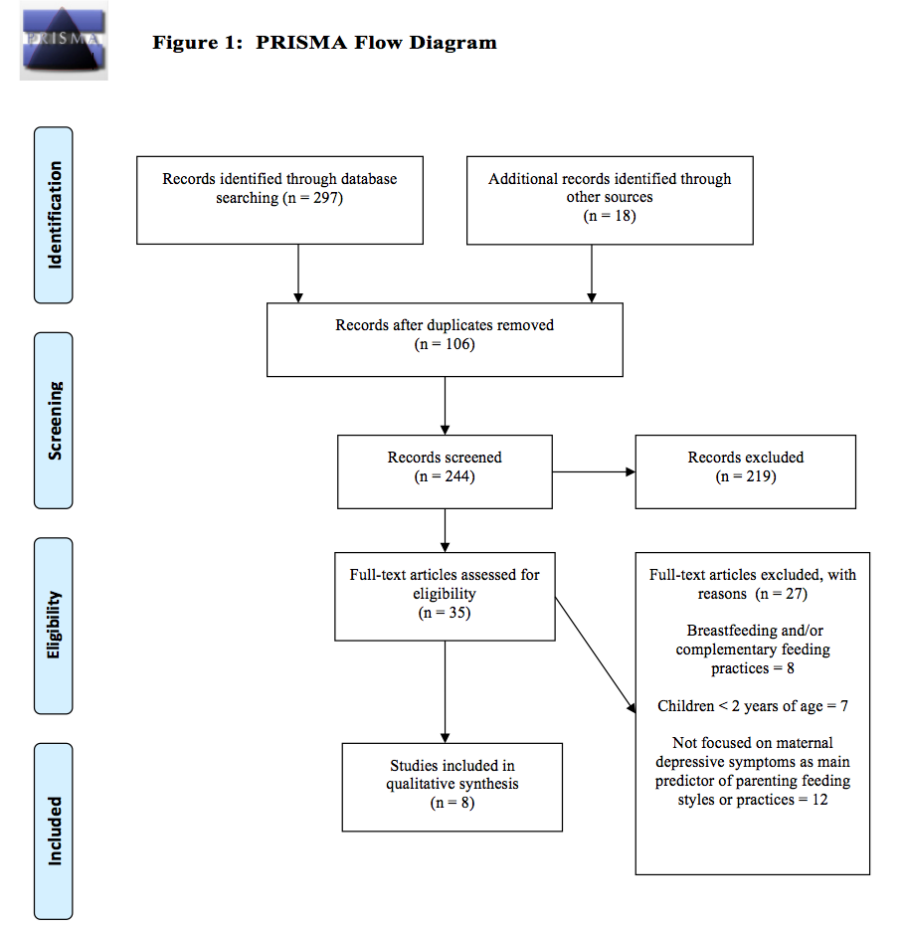
PRISMA Flow Diagram.

We searched six electronic databases: Science Direct, PubMed, PsycINFO, PscyARTICLES, Medline, and Cumulative Index to Nursing and Allied Health Literature (CINAHL) between April and June 21, 2016. The search was limited to full-text, peer-reviewed articles published in English between January 2000 and June 2016. Key search terms included: (1) child* OR pediatric*; (2) maternal depress* OR maternal depress* symptoms OR maternal “depressive symptoms”; and (3) “feeding practices” OR “feeding behavior” OR feeding srateg* OR feeding style (see Figure 1). Two authors (ACL, TM) independently examined the titles and abstracts of all citations, and the citations were excluded when both authors determined that the study did not meet the inclusion criteria. The same two authors independently reviewed the retrieved articles and identified studies to be included in this systematic review. They also searched the reference lists [39-46] and other review studies focusing on infants and/or children older than 8 years of age [7,10-16,25,31,47-49].

### Study Selection

This systematic review was limited to peer-reviewed studies that included mothers 18+ years old of normally developing children (ie, not born preterm, not diagnosed with physical or mental complications) between 2 and 8 years of ages (ie, early childhood). We identified studies that (1) examined the association between maternal depression and/or maternal depressive symptoms (independent variable) and parental feeding styles, parental feeding practices, and/or family mealtime practices (outcome variables), and (2) measured maternal depression or depressive symptoms with a validated questionnaire or scale at any period prior to or following childbirth. Studies that focused on special groups (eg, teen mothers, children born pre-term or low birth weight, or special needs such as cerebral palsy) or populations with health concerns (eg, mothers diagnosed with HIV) were excluded. Studies that used qualitative methods exclusively were also excluded to simplify comparison of findings across studies. Additionally, studies that focused exclusively on breastfeeding and/or complementary feeding practices were excluded as previous review papers have examined the association between maternal depressive symptoms and infant feeding practices [15,25,31,47,49].

### Data Extraction and Data Synthesis

Using the search strategy illustrated in Figure 1 (PRISMA flow diagram), we identified eight observational studies meeting eligibility requirements [39-46]. Two authors (ACL, TM) independently read and completed an article extraction form for all articles. The data extraction form gathered the following information: (1) authors, (2) study setting, (3) sample size, (4) participant characteristics, (5) study design, (6) study aim(s), (7) measure (s) of maternal depressive symptoms, (8) measure(s) of parental feeding styles and practices and family mealtime practices, and (9) study results. The two authors who completed the data extraction forms compared their results and discussed discrepancies, which were resolved with feedback from a third author.

This review extracted data on associations between maternal depressive symptoms (exposure) and parental feeding styles, parental feeding practices, and/or family mealtime practices (outcomes) and summarized the findings. Due to the range of study designs, assessment of exposure, and outcomes, conducting a meta-analysis of the data was not appropriate, and results of this review are presented as a narrative summary.

### Quality Assessment of Included Studies

The first author (ACL) assessed the quality of reviewed studies using a modified version of the Strengthening in the Reporting of Observational Studies in Epidemiology (STROBE) statement [50], which were confirmed by two authors (MLG, JAW). STROBE is an international, collaborative initiative of epidemiologists, methodologists, statisticians, researchers and journal editors who have a common goal of strengthening reporting of observational studies in epidemiology. The combined STROBE checklist for cohort, case-control, and cross-sectional studies includes 22 items [50]. For this review, we included 11 of these items to (1) identify potential sources of bias, and (2) identify possible methodological areas that were insufficiently addressed (see Multimedia Appendix 1). Each question was designed to be answered either “yes” or “no,” with a score of 1 assigned to “yes” response, and a score of 0 to “no” response (range of scores 0-11). Total scores were then used to assign a rating of the study as strong (score> 8), moderate (score between 8-6), or weak (score between 5-0).

## Results

### Search Results

We identified 297 unique citations and two authors (ACL, TM) independently examined the titles and abstracts of the identified citations. We excluded 272 citations that did not meet the eligibility criteria, and 35 full-text articles were selected for detailed review and assessed by two authors independently. Eight studies that met the eligibility criteria were included in this systematic review (see Figure 1). Studies included in this review fell into three categories, and this review is organized by the study’s purpose. The three purpose categories were to examine the associations between: (1) maternal depressive symptoms and nonresponsive parental feeding styles, (2) maternal depressive symptoms and nonresponsive parental feeding practices, and (3) maternal depressive symptoms and nonresponsive family mealtime practices.

### Study Characteristics

Eligible studies examined maternal depressive symptoms [51-56] and parental feeding styles [57] and feeding practices [58-64] using a wide variety of tools. Of the 8 included studies, 3 focused on feeding styles [39,41,46], 6 on nonresponsive feeding practices [40,41,43-46] and 3 on nonresponsive family mealtime practices [41,42,44]. Summary characteristics of included studies are presented in Multimedia Appendix 2, whereas synthesized information on methodology and main findings are presented in Multimedia Appendix 3.

### Study Quality

The quality of the reviewed studies varied (See Multimedia Appendix 1). Out of the 8 included studies, 6 were assigned a rating of > 8 (strong), and 2 were rated between 8-6 (moderate). Most of the reviewed studies presented methodological limitations: (1) nearly all (6/8) used cross-sectional study designs that prohibit determining causal inferences between maternal depressive symptoms and child-feeding practices and styles [39,41,42,44-46], (2) all studies (8/8) relied on self-reporting of maternal depressive symptoms [39-46], and (3) the majority (6/8) relied on self-reporting of parental feeding styles, parental feeding practices and family mealtime practices [39-42,44,46]. Furthermore, it was not always possible to determine whether analyses controlled for potential confounding factors and analyses were not stratified by child gender.

Moreover, maternal depressive symptoms [51-56] and parental feeding practices [58-64] were assessed by an array of instruments, which made it difficult to compare results across studies. For example, across the 8 reviewed studies, 6 different instruments were used to measure maternal depressive symptoms [51-56], whereas 7 instruments were used to assess parental feeding practices [58-64], 2 instruments were used to assess family mealtime practices [59,61], and 1 to assess parental feeding styles [57].

### Associations Between Maternal Depressive Symptoms and Nonresponsive Feeding Styles

Out of the 8 included studies [39-46], 3 studies [39,41,46] examined the association between maternal depressive symptoms and nonresponsive feeding styles. Two [39,41] of these studies used the validated Center for Epidemiological Study Depression (CES-D) instrument [51], whereas 1 used the Depression Anxiety Stress Scale 21-item (DASS-21) [53] to assess maternal depressive symptoms. All three studies were cross-sectional [39,41,46] and used the validated Caregiver’s Feeding Styles Questionnaire (CFSQ) [57] to assess parental feeding styles. The CFSQ is a 19-item valid, reliable measure developed for use with caregiver’s of preschool-age children [57].

Of the 3 studies examining associations between maternal depressive symptoms and nonresponsive parental feeding styles in adjusted analysis, only 2 studies found positive associations [39,41], with mothers with elevated depressive symptoms being more likely to exhibit uninvolved [39,41] and permissive styles [41] than those without these symptoms.

A cross-sectional study [41] conducted in the United States with a sample of low-income mothers of whom approximately 30% were Hispanics, found that mothers reporting elevated depressive symptoms reported using more demandingness (eg, encouragement or discouragement of child’s eating behaviors) and permissive feeding styles (eg, fewer authority narratives about feeding) than mothers without elevated depressive symptoms after adjusting for potential confounders. One cross-sectional study [39] conducted in the United States with a sample of low-income mothers, of whom approximately 55% were Hispanic and 45% were African-American, found that after adjusting for potential confounders, mothers employing an uninvolved feeding style (a permissive feeding style) reported less positive affect and more parenting stress than mothers who used authoritative, authoritarian, or indulgent/permissive feeding styles. In addition, mothers with elevated depressive symptoms were more likely to present low authority in child feeding [39].

One cross-sectional study [46] conducted in Australia found that mothers who reported experiencing higher levels of depressive symptoms also reported using higher levels of the authoritarian feeding style. In adjusted analysis, however, none of the maternal psychosocial well-being variables independently contributed to the prediction of authoritarian parental feeding style.

### Associations Between Maternal Depressive Symptoms and Nonresponsive Feeding Practices

Out of the 8 reviewed studies, 6 [40,41,43-46] examined the association between maternal depressive symptoms and nonresponsive feeding practices. Four studies [41,44-46] employed cross-sectional designs and two used longitudinal designs [40,43]. The 6 studies used five different instruments [51,53-56] to assess maternal depressive symptoms, with the Edinburgh Postnatal Depression Scale (EPDS) [53] being used in 2 [40,43] and the Depression Anxiety Stress Scales 21-item (DASS-21) [54] also being used in 2 studies [43,46]. The validated Child Feeding Questionnaire (CFQ) [58] was the instrument most used to assess parental feeding practices (5/6). In addition, five other validated instruments were used across the 6 studies [59-64]. All 6 reviewed studies [40,41,43-46] provided information on the reliability and validity of the instruments used to assess both maternal depressive symptoms and parental feeding practices (see Multimedia Appendix 3).

In summary, 3 studies reported positive associations between maternal depressive symptoms and use of instrumental feeding (eg, using food as a reward) (3/3) [40,44,45], and pressure to eat (3/6) [40,41,45]. One study reported positive associations between maternal depressive symptoms and restriction of child’s food intake (1/6) [40], and emotional (eg, using food to manage child’s mood) feeding (1/3) [40]. In addition, 2 studies (2/6), one with a cross-sectional design [44] and one using a longitudinal design [43], reported negative associations between maternal depressive symptoms and restriction of child food intake. In contrast, a cross-sectional study (1/6) [45] found that elevated depressive symptoms were not associated with restriction of child food intake. Moreover, 1 study (1/4) reported that elevated depressive symptoms were negatively associated with monitoring of child food intake [43].

#### Pressure to Eat

Six of the reviewed studies examined the association between maternal depressive symptoms and mothers’ use of pressure to get their children to eat [40,41,43-46], and all found positive associations between elevated maternal depressive symptoms and pressure to eat in unadjusted analyses. However, only three studies (3/6), one using a longitudinal design [40] and two using cross-sectional designs [41,45], reported significant positive associations between maternal depressive symptoms and pressure to eat after adjusting for several key child (eg, age, gender, child body mass index) and maternal characteristics (eg, age, BMI, race, income, educational level). Results of these 3 studies [40,41,45] indicated that mothers reporting elevated depressive symptoms were more likely to report pressuring their children to eat than mothers without elevated depressive symptoms after adjusting for potential confounding factors.

#### Restriction of Child’s Food Intake

Six of the reviewed studies [40,41,43-46] examined associations between maternal depressive symptoms and restrictions in child food intake. Three of these studies [40,41,46] reported positive associations between maternal general depressive symptoms and restriction in feeding in unadjusted analyses. However, after adjusting for key maternal (age, education, BMI) and child covariates (age, gender, BMI at 4 months and feeding mode at 4 months), only one longitudinal study [40] found that maternal general depressive symptoms were associated with the restriction of children’s food intake. In contrast, a longitudinal study [43] found that high depressive symptoms predicted less maternal use of restriction. Likewise, a study [44] using a cross-sectional design found a negative association between maternal depressive symptoms and use of restriction of child’s food intake, with mothers reporting mild and moderate to severe symptoms were less likely to restrict their child’s intake than mothers not reporting depressive symptoms. Moreover, a cross-sectional study [45] determined that maternal depressive symptoms were not predictive of mothers’ restrictive feeding practices.

#### Monitoring of Child Food Intake

Four of the reviewed studies, two employing longitudinal study design [40,43] and two cross-sectional designs [41,44], examined associations between maternal depressive symptoms and monitoring of child food intake. Of the 4 studies, 1 longitudinal follow-up study found that maternal depressive symptoms partially negatively predicted monitoring of child food intake [43].

#### Instrumental and Emotional Feeding Practices

Three [40,44,45] studies examined the relationship between maternal depressive symptoms and instrumental feeding practices (eg, using food as a reward, increased use of incentives) and emotional feeding (eg, using food to manage child mood). All 3 studies found a positive association between maternal depressive symptoms and instrumental feeding [40,44,45], whereas one (1/3) found a positive association between maternal depressive symptoms and emotional feeding [40]. One longitudinal study found that mothers with elevated depressive symptoms were more likely to employ both instrumental and emotional feeding practices adjusting for maternal and child covariates [40] than mothers with low or without depressive symptoms. Additionally, 2 studies using cross-sectional designs [44,45], one conducted in the United States [44], and one in England [45], found positive associations between maternal depressive symptoms and the use of instrumental feeding practices (eg, use of food as reward, or use of incentive and conditions to get child to eat). In adjusted analysis, higher maternal depressive symptoms were significantly associated with use of food as a reward [44] and with greater use of incentives or conditions to eat [45].

### Associations Between Maternal Depressive Symptoms and Nonresponsive Family Mealtime Practices

Out of the 8 reviewed studies [39-46] 3 [41,42,44], all of which employed cross-sectional designs and were conducted in the United States with low-income mothers, examined the association between maternal depressive symptoms and a number of nonresponsive family mealtime practices. Two [41,42] of these studies used the Center for Epidemiological Study Depression (CES-D) instrument [51], whereas one [44] used the Patient Health Questionnaire-9 (PHQ-9) [55] to assess maternal depressive symptoms. Two different instruments [59,61] were used to assess family mealtime practices by 2 [42,44] of the 3 studies, and 1 study [41] used both semistructured narrative interview and videotaped observations of mother-child feeding situations.

All 3 studies determined that children in households with mothers with elevated depressive symptoms were more likely to be exposed to less optimal mealtime practices and routines than children in households with mothers with low or without any depressive symptoms [41,42,44]. Moreover, all 3 studies found that mothers reporting elevated depressive symptoms were more likely to report nonresponsive feeding practices which were associated with both uninvolved (eg, mother not being present during meals, child skipping breakfast, child eating while watching television) and permissive (eg, lower levels of maternal control over child eating routines, greater child choice over snacking) feeding styles.

One study [41] found that in households of mothers with elevated depressive symptoms, children were less likely to eat at the kitchen or dining table, the television was more likely to be audible during meals, and children were less likely to eat with their mothers. Similarly, 1 study [42] found that maternal depression was significantly associated with lower maternal presence when the child ate, lower levels of maternal control over child eating routines, greater child choice over snacking, and fewer optimal mealtime practices than in homes of mothers without higher depression scores [42]. Likewise, 1 study [44] found in adjusted analyses that mothers reporting mild depressive symptoms were more likely to have children who consumed sweetened drinks daily, who did not eat breakfast regularly, and who ate out in restaurants 3 or more times per week than mothers without depressive symptoms.

## Discussion

### Principal Findings

The aim of this systematic review was to identify and review existing research examining associations between maternal depressive symptoms and nonresponsive parental feeding styles and parental feeding practices in mothers of young children. The 8 reviewed studies provide mixed support for associations between maternal depressive symptoms and nonresponsive feeding styles, feeding practices, and family mealtime practices. Uninvolved and permissive feeding styles, and feeding practices use of instrumental feeding (eg, use of food as reward) and pressuring children to eat were the most consistently associated with depressive symptoms among studies included in this review. In addition, maternal depressive symptoms were associated with uninvolved and permissive family mealtime practices.

Across the reviewed studies, elevated maternal depressive symptoms were most often associated with uninvolved and permissive parental feeding styles [39,41]. Two of the three studies examining associations between maternal depressive symptoms and nonresponsive feeding styles (uninvolved and permissive) found a positive association [39,41]. These findings concur with results of studies [11,12] and review papers [10,15,25,47,49,65] conducted among mothers with infants. Furthermore, available evidence from the extant literature on maternal mental health and parenting suggests that maternal mental health issues may impair mothers’ responsiveness to, and interactions with, their children. The reduced interaction may manifest in nonresponsive, more controlling, and less-sensitive parenting [10,29,37,47,49,60]. In addition, research suggest that elevated depressive symptoms may contribute to decreased maternal–child interactions [30,66], with mothers being less responsive to their children and choosing strategies for coping that require less cognitive effort [11-13].

Evidence for associations between maternal depressive symptoms and nonresponsive maternal feeding practices was mixed across reviewed studies [40,41,43-46]. Instrumental feeding (eg, use of food as reward; 3/3) [40,44,45] and pressure to eat (3/6) [40,41,45] were the most consistently nonresponsive feeding practices associated with elevated depressive symptoms across the studies included in this review. Evidence from studies [11,12,16] and systematic reviews [25,47] with mothers of infants and toddlers suggests that mothers experiencing elevated depressive symptoms are more likely to use restrictive and controlling feeding practices than mothers without elevated depressive symptoms. Moreover, evidence suggest that mothers experiencing elevated depressive symptoms are less likely to be responsive to their children’s cues of hunger and satiety and less likely to respect their child’s ability to self-regulate food intake [10-13,66]. Previous studies indicate that nonresponsive feeding practices interfere with a child’s natural ability to self-regulate food intake based on hunger and satiety cues [14,19,30,67-69]. Furthermore, research suggests that both parental pressure to eat and feeding restrictions are associated with unrestrained eating and disinhibited eating in later life, excessive weight gain, and increased risk of child obesity in children [5,20,21,67-71].

Studies included in this review provide consistent evidence for the association between maternal depressive symptoms and nonresponsive family mealtime practices [41,42,44]. Children in households with mothers having elevated depressive symptoms were more likely to be exposed to less optimal mealtime practices and routines than children in households with mothers having low or no depressive symptoms [41,42,44]. Elevated depressive symptoms such as low energy and diminished pleasure in activities may contribute to decreased maternal involvement with the child [5,21,67-69,30], resulting in mothers being less responsive to their children and choosing strategies for coping that require less cognitive effort [5]. Suboptimal family mealtime practices have been reported to be associated with children’s unhealthy eating habits [72-77], which in turn have been linked to risk of overweight and obesity [72-77].

### Limitations and Strengths

Our evaluation of the methodologies of studies included in this systematic review suggests some limitations, and therefore caution in the interpretation of study findings. The majority (6/8) of studies used cross-sectional study designs precluding a causal assessment of associations between maternal depressive symptoms and feeding styles and practices [39,41,42,44-46]. Additional longitudinal studies are necessary to understand whether mothers’ depressive symptoms influence their feeding styles and practices. Furthermore, nearly all examined (6/8) studies used an array of self-reported questionnaires for assessments of maternal depressive symptoms and parental feeding practices (7/8), which is potentially problematic due to possible misclassification of depressive symptoms (exposure) and parental feeding practices (outcome). Finally, variability in the assessment of maternal depressive symptoms (eg, CES-D, DASS, BSI) and parental feeding practices (eg, Child Feeding Styles Questionnaire [CFSQ], CFQ, Family Mealtime Coding System [FMCS]) make it difficult to compare findings across studies and indicate that caution must be taken in drawing conclusive assertions.

Strengths of this review include the use of systematic criteria (ie, PRISMA) to identify and select studies and a quality assessment tool for the critical appraisals of studies. Nonetheless, this review may be incomplete given limitation to studies published in English. Another possible limitation of this review is the variability in the studies’ location. Multiple countries (United States, Australia, and England) were represented, which may limit cross-study comparisons. Finally, publication bias should also be considered, as should the fact that this review is limited to full-text studies published in English and may have excluded studies published in other formats and/or languages.

### Future Directions

Additional research is needed to further examine the relationships between maternal depressive symptoms and nonresponsive parental feeding styles and practices. Specifically, longitudinal studies and additional studies including low-income and racial/ethnic minority populations at increased risk of depressive symptoms are needed. Future studies should explore the associations between maternal depressive symptoms, food insecurity and maternal feeding styles and practices. This is required especially due to documented evidence of greater prevalence of obesity among racial/ethnic minority populations [78]. In 2 examined studies [39,42], authors suggest that food insecurity may interact with maternal depressive symptoms such as stress to increase the risk of unintentionally detrimental feeding practices such as pressuring child to eat and/or restricting child food intake. Therefore, studies that assess the potential interactions of food security status and maternal depressive symptoms on maternal feeding styles and practices are needed. Moreover, given the inconsistencies in results across studies included in this review, future research should also consider the potential influence of additional factors such as mother’s socioeconomic status, acculturation level, social support, as well as contextual factors such as work strain, access to healthful foods, and so on. Finally, future research may benefit from examining differentials of depressive symptoms and parental feeding styles and practices according to the gender of the parent and the child.

### Conclusions

In summary, studies identified and synthesized in this review provided mixed evidence for associations between maternal depressive symptoms and nonresponsive maternal feeding styles and practices. Nevertheless, given the high prevalence of maternal depressive symptoms among women of reproductive age [32-35], the indication from some studies of associations between maternal depressive symptoms and nonresponsive feeding styles and practices, and the fact that both maternal depressive symptoms and that nonresponsive feeding styles and practices are potentially modifiable, further understanding of these associations are likely to provide important insights for the development of interventions to prevent and control childhood obesity.

## Appendix

Multimedia Appendix 1 Quality assessment of 8 included studies using an adapted version of the Strengthening the Reporting of Observational Studies in Epidemiology (STROBE).

Multimedia Appendix 2 Description of the 8 studies included in systematic review.

Multimedia Appendix 3 Characteristics of 8 included studies included in systematic review.

## Abbreviations

BMI: body mass index
BSI: Brief Symptom Inventory
CES-D: Center for Epidemiologic Studies Depression Scale
CFSQ: Child Feeding Styles Questionnaire
CFQ: Child Feeding Questionnaire
CINAHL: Cumulative Index to Nursing and Allied Health Literature
DASS: Depression Anxiety Stress Scales
EPDS: Edinburgh Postnatal Depression Scale
FMCS: Family Mealtime Coding System
HIV: human immunodeficiency virus
PRISMA: Preferred Reporting Items for Systematic Reviews and Meta-Analysis
STROBE: Strengthening in the Reporting of Observational Studies in Epidemiology
