# Supplementary material for: Associations Between Maternal Depressive Symptoms and Nonresponsive Feeding Styles and Practices in Mothers of Young Children: A Systematic Review
Source: JMIR Public Health Surveill. 2017 May 26;3(2):e29. doi: 10.2196/publichealth.6492 (PMC5466702; doi:10.2196/publichealth.6492)
Supplement: Multimedia Appendix 1 [file publichealth_v3i2e29_app1.pdf]

Multimedia Appendix 1: Quality assessment of 8 included studies using an adapted version of the Strengthening the Reporting of Observational Studies in Epidemiology (STROBE)

Items:
#1. Did the study employ a longitudinal cohort design study?
#2. Did the paper describe the participants' eligibility criteria?
#3. Were the participants randomly selected (or representative of the study population)?
#4. Did the paper report the sources and details of assessment of depressive symptoms?
#5. Did the instruments scales (depressive symptoms) have acceptable validity and reliability?
6. Did the paper report the sources and details of assessments of maternal feeding practices and/or styles?
#7. Did the instruments scales (child feeding styles and/or practices) have acceptable validity and reliability?
#8. Was the study adequately powered to detect hypothesized relationships?
#9. Did the paper report the number of individuals who completed each of the different measures?
#10. Did the participants complete at least 80% of measures?
#11. Did statistical analysis take into account confounding factors?

Studies	#1	#2	#3	#4	#5	#6	#7	#8	#9	#10	#11	Total
Hughes et al. [39], USA	0	1	0	1	1	1	1	1	1	1	1	9
Mallan et al. [40], Australia	1	1	1	1	1	1	1	1	1	1	1	11
Goulding et al. [41], USA	0	1	0	1	1	1	1	1	1	1	1	9
McCurdy et al. [42], USA	0	1	0	1	1	1	1	1	1	1	1	9
Gemmill et al. [43], Australia	1	1	1	1	1	1	1	1	1	0	1	10
Gross et al. [44], USA	0	1	0	1	1	1	1	1	1	1	1	9
Haycraft & Farrow [45], England	0	0	0	1	1	1	1	0	1	1	1	7
Mitchell et al. [46], Australia	0	1	0	1	1	1	1	0	1	1	1	8

References

39. Hughes SO, Power TG, Liu Y, Sharp C, Nicklas TA. Parent emotional distress and feeding styles in low-income families: the role of parent depression and parenting stress. Appetite. 2015 Sep; 92:337-42. doi: 10.1016/j.appet.2015.06.002.
40. Mallan KM, Daniels LA, Wilson JL, Jansen E, Nicholson JM. Association between maternal depressive symptoms in the early post-natal period and responsiveness in feeding at child age 2 years. Matern Child Nutr. 2015 Oct;11(4):926-35. doi: 10.1111/mcn.12116.
41. Goulding AN, Rosenblum KL, Miller AL, Peterson KE, Chen YP, Kaciroti N, Lumeng JC. Associations between maternal depressive symptoms and child feeding practices in a cross-sectional study of low-income mothers and their young children. Int J Behav Nutr Phys Act. 2014 Jun 16;11:75. doi: 10.1186/1479-5868-11-75.
42. McCurdy K, Gorman KS, Kisler T, Metallinos-Katsaras E. Associations between family food behaviors, maternal depression, and child weight among low-income children. Appetite. 2014 Aug;79:97-105. doi: 10.1016/j.appet.2014.04.015.
43. Gemmill AW, Worotniuk T, Holt CJ, Skouteris H, Milgrom J. Maternal psychological factors and controlled child feeding practices in relation to child body mass index. Child Obes. 2013 Aug;9(4):326-37. doi: 10.1089/chi.2012.0135.

44. Gross RS, Velazco NK, Briggs RD, Racine AD. Maternal depressive symptoms and child obesity in low-income urban families. Acad Pediatr. 2013 Jul-Aug;13(4):356-63. doi: 10.1016/j.acap.2013.04.002.
45. Haycraft E, Farrow C, Blissett J. Maternal symptoms of depression are related to observations of controlling feeding practices in mothers of young children. J Fam Psychol. 2013 Feb;27(1):159-64. doi: 10.1037/a0031110.
46. Mitchell S, Brennan L, Hayes L, Miles CL. Maternal psychosocial predictors of controlling parental feeding styles and practices. Appetite. 2009 Dec;53(3):384-9. doi: 10.1016/j.appet.2009.08.001.
