# Supplementary material for: Associations Between Maternal Depressive Symptoms and Nonresponsive Feeding Styles and Practices in Mothers of Young Children: A Systematic Review
Source: JMIR Public Health Surveill. 2017 May 26;3(2):e29. doi: 10.2196/publichealth.6492 (PMC5466702; doi:10.2196/publichealth.6492)
Supplement: Multimedia Appendix 2 [file publichealth_v3i2e29_app2.pdf]

Multimedia Appendix 2: Description of the 8 studies included in systematic review.

Characteristics	No. of Studies
**Total number of studies selected**	8
**Publication dates**	
2000 – 2005	0
2006 – 2010 [46]	1
2011 – 2016 [39-45]	7
**Study design**	
Cohort/longitudinal [40, 43]	2
Cross-sectional [39, 41, 42, 44, 45, 46]	6
**Children's age groups**	
2 – 5 years of age [39, 40, 42, 44, 45]	5
2 – 7 years of age [43]	1
4 – 8 years of age [41, 46]	2
**Countries represented**	
U.S.A [39, 41, 42, 44]	4
England [45]	1
Australia [40, 43, 46]	3
**Assessment of maternal depressive symptoms**	
Center for Epidemiologic Studies Depression scale (CES-D) [39, 45, 46]	3
Parent Stress Index–Short Form (PSI-SF) [39]	1
Edinburgh Postnatal Depression Scale (EPDS) [40, 43]	2
Patient Health Questionnaire-9 (PHQ-9) [44]	1
Hospital Anxiety and Depression Scale (HADS) [45]	1
Depression Anxiety Stress Scales 21-item (DASS-21) [43, 46]	2
**Assessment of child feeding styles and practices**	
Child Feeding Styles Questionnaire (CFSQ) [39, 40, 46]	3
Child Feeding Questionnaire (CFQ) [40, 41, 43, 44, 46]	5
Family Food Behavior Survey (FFBS) [46]	1
Parental Feeding Style Questionnaire (PFSQ) [40]	1
Comprehensive Feeding Practices Questionnaire (CFPQ) [44]	1
Parenting Strategies for Eating and Activity Scale (PEAS) [44]	1
Mealtime Coding System (MCS) [45]	1
Overt/Covert Control Scale (OCCS) [46]	1
Semi-structured narrative interview [40]	1
Videotaped observations of mother-child feeding situations [40, 45]	2

References

39. Hughes SO, Power TG, Liu Y, Sharp C, Nicklas TA. Parent emotional distress and feeding styles in low-income families: the role of parent depression and parenting stress. Appetite. 2015 Sep; 92:337-42. doi: 10.1016/j.appet.2015.06.002.

40. Mallan KM, Daniels LA, Wilson JL, Jansen E, Nicholson JM. Association between maternal depressive symptoms in the early post-natal period and responsiveness in feeding at child age 2 years. Matern Child Nutr. 2015 Oct;11(4):926-35. doi: 10.1111/mcn.12116.
41. Goulding AN, Rosenblum KL, Miller AL, Peterson KE, Chen YP, Kaciroti N, Lumeng JC. Associations between maternal depressive symptoms and child feeding practices in a cross-sectional study of low-income mothers and their young children. Int J Behav Nutr Phys Act. 2014 Jun 16;11:75. doi: 10.1186/1479-5868-11-75.
42. McCurdy K, Gorman KS, Kisler T, Metallinos-Katsaras E. Associations between family food behaviors, maternal depression, and child weight among low-income children. Appetite. 2014 Aug;79:97-105. doi: 10.1016/j.appet.2014.04.015.
43. Gemmill AW, Worotniuk T, Holt CJ, Skouteris H, Milgrom J. Maternal psychological factors and controlled child feeding practices in relation to child body mass index. Child Obes. 2013 Aug;9(4):326-37. doi: 10.1089/chi.2012.0135.
44. Gross RS, Velazco NK, Briggs RD, Racine AD. Maternal depressive symptoms and child obesity in low-income urban families. Acad Pediatr. 2013 Jul-Aug;13(4):356-63. doi: 10.1016/j.acap.2013.04.002.
45. Haycraft E, Farrow C, Blissett J. Maternal symptoms of depression are related to observations of controlling feeding practices in mothers of young children. J Fam Psychol. 2013 Feb;27(1):159-64. doi: 10.1037/a0031110.
46. Mitchell S, Brennan L, Hayes L, Miles CL. Maternal psychosocial predictors of controlling parental feeding styles and practices. Appetite. 2009 Dec;53(3):384-9. doi: 10.1016/j.appet.2009.08.001.
