# Supplementary material for: Associations Between Maternal Depressive Symptoms and Nonresponsive Feeding Styles and Practices in Mothers of Young Children: A Systematic Review
Source: JMIR Public Health Surveill. 2017 May 26;3(2):e29. doi: 10.2196/publichealth.6492 (PMC5466702; doi:10.2196/publichealth.6492)
Supplement: Multimedia Appendix 3 [file publichealth_v3i2e29_app3.pdf]

Multimedia Appendix 3: Characteristics of 8 included studies included in systematic review.

Authors (reference), Setting	Sample size Participant Characteristics; Study Design	Study Aim (s)	Measures of Maternal Depressive Symptoms	Measures of Maternal Feeding Styles and/or Practices	Main Findings
Hughes et al. (2015) [39] USA	n = 290 Mean mother age = 31.5 ± 7.5 years Mean child age = 4.43 ± 0.7 years (range 2-5 years) 54.8% Hispanic; 45.8% African American Cross-sectional	To examine the relationships between parental emotional distress and parent feeding. The specific aim was to examine how depressive symptoms and parenting stress might influence the nature of parent feeding styles.	Center for Epidemiologic Studies Depression scale (CES-D) [51] and Parent Stress Index–Short Form (PSI-SF) [52].	Demandingness and responsiveness scales in the Caregiver's Feeding Styles Questionnaire (CFSQ) [57] to generate four parenting feeding styles: authoritative, authoritarian, indulgent, and uninvolved.	Uninvolved parents were more likely to report higher parenting stress and less likely to report positive affect than parents showing the other three feeding styles (authoritative, authoritarian, and indulgent).
Mallan et al. (2014) [40] Australia	n = 211 Mean mother age = 30 ± 5 years (at delivery) Mean child age = 24.1 ± 0.7 months Majority White Longitudinal	To investigate whether level of post-natal depressive symptomatology (baseline: child age 4 months) predicts non-responsive feeding practices when children are 2 years of age, after adjusting for key covariates (measured at baseline including both maternal characteristics (age, BMI, education level, and breastfeeding status) and infant characteristics (gender, temperament, and BMI z-score).	Edinburgh Postnatal Depression Scale (EPDS) [53].	The Child Feeding Questionnaire (CFQ) [58] subscales pressure to eat, restriction and monitoring. The Parental Feeding Style Questionnaire (PFSQ) [62].	Higher EPDS score was associated with less responsive feeding practice at child age 2 years: greater pressure (β = 0.18, 95% CI: 0.04-0.32, p = 0.01), restriction (β = 0.14, 95% CI: 0.001-0.28, p = 0.05), instrumental (β = 0.14, 95% CI: 0.005-0.27, p = 0.04), and emotional feeding (β = 0.15 95% CI: 0.01-0.29, P = 0.03) feeding practices ($\Delta R(2)$ values: 0.02-0.03, $p < 0.05$).

**Authors (reference), Setting**	**Sample size Participant Characteristics; Study Design**	**Study Aim (s)**	**Measures of Maternal Depressive Symptoms**	**Measures of Maternal Feeding Styles and/or Practices**	**Main Findings**
Goulding et al. (2014) [41] USA, Michigan	n=295<br>Mean mother age = 30.99 ± 7.03 years<br>Mean child age = 70.83 ± 8.32 months (range: 4-8 years) 32% Hispanic<br>Cross-sectional	To examine associations between maternal depressive symptoms and child feeding practices in a population of low-income mothers of 4- to 8-year-old children.<br>To test the hypothesis that mothers with elevated depressive symptoms exhibit less responsive feeding practices than mothers with lower levels of depressive symptoms.	CES-D [51].	The CFQ's [58] subscales monitoring, pressure to eat, perceived responsibility, weight, perceived child weight, concern about child, and restriction.<br>The Demandingness scale in the CFSQ [57].<br>Semi-structured narrative interview. Interviews were designed to elicit maternal narrative about feeding.<br>Videotaped observations of mother-child feeding situations. For the home meal observations, each mother was loaned a camera and asked to videotape three typical dinnertime meals over the course of one week.	Mothers with elevated depressive symptoms reported more pressuring of children to eat ($\beta = 0.29$; 95% CI: 0.03, 0.54), more overall demandingness ($\beta = 0.16$; 95% CI: 0.03, 0.29), and expressed lower authority in child feeding during semi-structured interview (OR for low authority: 2.82; CI: 1.55, 5.12).<br>In homes of mothers with elevated depressive symptoms, the television was more audible during meals (OR: 0.48; 95% CI: 0.27, 0.85). There were associations between maternal depressive symptoms and encouragement or discouragement of food in laboratory eating interactions.
McCurdy et al. (2014) [42]<br>USA	n = 164<br>Mean mother age = 30.1 ± 7.2 years<br>Mean child age = 51.4 ± 10.1 months<br>55% Hispanic<br>Cross-sectional	To examine associations between family food behaviors (including family mealtime practices and food resource management skills), maternal depression, and child weight in a sample of ethnically diverse, low-income families.	CES-D [51].	The 20-item Family Food Behavior Survey (FFBS) [59].	Multivariate analysis of covariance revealed that higher maternal depression scores were associated with lower scores of maternal presence when child eats ($p < 0.05$), maternal control of child's eating routines ($p < 0.03$), and negative mealtime practices ($p < 0.5$).

Authors (reference), Setting	Sample size Participant Characteristics; Study Design	Study Aim (s)	Measures of Maternal Depressive Symptoms	Measures of Maternal Feeding Styles and/or Practices	Main Findings
Gemmill et al. (2013) [43] Australia	n = 203<br>Mean mother age = 33.96 ± 4.82 years (T1)<br>Mean child age = 4.82 ± 0.8 years<br>90.9% White<br>Longitudinal	To examine whether controlling maternal feeding practices predicts child BMI, and to investigate whether maternal depressive and anxious symptoms are predictive of child feeding practices.	Edinburgh Postnatal Depression Scale (EPDS) [53]; Depression Anxiety Stress Scales 21-item (DASS-21) [54].	The CFQ's subscales pressure to eat, restriction, and monitoring [58].	Maternal use of restriction and monitoring were partially positively predicted by concurrent maternal stress and negatively partially predicted by concurrent depression.<br>Mothers enduring high stress appeared to employ more controlled feeding patterns, whereas mothers experiencing depression seemingly employed lower levels of controlled feeding.
Gross et al. (2013) [44] USA	n = 401<br>Mean mother age = 32.8 ± 6.1 years<br>Child age = 5 years (mean and standard deviation was not provided)<br>49.9% Hispanics;<br>34.4% Black;<br>4.2% Asian<br>Cross-sectional	To characterize the relationship between maternal depressive symptoms and early childhood weight as well as the relationship of these symptoms with obesity-promoting behaviors in low-income minority families with 5- year-old children.	Patient Health Questionnaire-9 (PHQ-9) [55].	The CFQ's subscales pressure to eat and restriction [58].<br>The Comprehensive Feeding Practices Questionnaire (CFPQ) [60] subscales monitoring food as reward, modeling, involvement, healthy eating environment and emotional regulation.<br>The Parenting Strategies for Eating and Activity Scale [61] subscales limit setting, monitoring, and reinforcement.	Mothers with moderate to severe depressive symptoms were more likely to have overweight and obese children than mothers without depressive symptoms (adjusted odds ratio: 2.62; 95% CI: 1.02-6.70).<br>Children of mildly depressed mothers were more likely to consume sweetened drinks and to eat out at restaurants, and were less likely to eat breakfast than children of non-depressed mothers.<br>Mothers with depressive symptoms were less likely to: set limits; use food as a reward; restrict their child's intake; and model healthy eating.

Authors (reference), Setting	Sample size Participant Characteristics; Study Design	Study Aim (s)	Measures of Maternal Depressive Symptoms	Measures of Maternal Feeding Styles and/or Practices	Main Findings
Haycraft & Farrow (2013) [45]<br>England	n = 58<br>Mean mother age = 34 ± 5.48 years<br>Mean child age = 3.8 ± 0.58 years<br>91% White<br>Cross-sectional	To examine relationships between self-reported maternal symptoms of depression with observations of mothers' child feeding practices during a mealtime in a laboratory setting.	Hospital Anxiety and Depression Scale (HADS) [56].	Video recorded laboratory observation coded using the Family Mealtime Coding System (MCS) [45] subscales verbal pressure to eat, physical pressure to eat, verbal restriction of food, physical restriction of food, and use of incentives/conditions.	Higher levels of depressive symptoms were related to greater use of verbal and physical pressure for children to eat, increased use of incentives, and a higher incidence of vocalizations about food during the observed meal.<br>Symptoms of depression were not significantly linked to observations of maternal restriction of food.<br>Symptoms of depression were linked with observation of mothers implementing a more controlling, less sensitive feeding style with their child.
Mitchell et al. (2009) [46]<br>Australia	n = 124<br>Mean mother age = 36.8 ± 4.62 years<br>Mean child age = 6.46 ± 0.95 years<br>89% White<br>Cross-sectional	To explore the relative contribution of parental depression, anxiety and stress, and parenting satisfaction and efficacy to the explanation of variance in controlling parental feeding styles and practices.	DASS-21 [54].	The CFQ's subscales pressure to eat and restriction [58].<br>The Overt/Covert Control Scale [63].	Authoritarian maternal feeding style was positively correlated with maternal depression, anxiety, and stress.<br>Restriction was positively correlated with maternal depression, anxiety, and stress.<br>Pressure to eat was positively correlated with maternal depression, anxiety, and stress.<br>In multivariate analysis, maternal anxiety and maternal satisfaction contributed significantly to the prediction of restriction. These two variables accounted for 12% of the variance in restriction, and overall model was significant $F(5, 114) = 4.10$, $p < .01$).

References

45. Haycraft E, Farrow C, Blissett J. Maternal symptoms of depression are related to observations of controlling feeding practices in mothers of young children. J Fam Psychol. 2013 Feb;27(1):159-64. doi: 10.1037/a0031110.
51. Radloff LS. The CES-D scale. A self-report depression scale for research in the general population. Appl Psychol Meas. 1977;1(3):385-401.
52. Abidin RR. Parenting stress index: professional manual. 3rd ed. Odessa, FL: Psychological Assessment Resources; 1995.
53. Cox JL, Holden JM, Sagovsky R. Detection of postnatal depression: development of the 10-item Edinburgh Postnatal Depression Scale. Br J Psychiatry. 1987 Jun;150:782-6.
54. Antony MM, Bieling PJ, Cox BJ, Enns MW, Swinson RP. Psychometric properties of the 42-item and 21-item versions of the Depression Anxiety Stress Scales in clinical groups and a community sample. Psychol Assess. 1998;10(2):176-81.
55. Kroenke K, Spitzer RL, Williams JB. The PHQ-9: validity of a brief depression severity measure. J Gen Intern Med. 2001;16:606-13.
56. Zigmond AS, Snaith RP. The hospital anxiety and depression scale. Acta Psychiatr Scand. 1983;67(6):361-70.
57. Hughes SO, Power TG, Fisher JO, Mueller S, Nicklas TA. Revisiting a neglected construct. Parenting styles in a child-feeding context. Appetite. 2005;44(1):83-92.
58. Birch LL, Fisher J, Grimm-Thomas K, Markey C, Sawyer R, Johnson S. Confirmatory factor analysis of the child feeding questionnaire: a measure of parental attitudes, beliefs and practices about child feeding and obesity proneness. Appetite. 2001;36(3):201-10.
59. McCurdy K, Gorman K. Measuring family food environments in diverse families with young children. Appetite. 2010;54:615-8.
60. Musher-Eizenman D, Holub S. Comprehensive feeding practices questionnaire: validation of a new measure of parental feeding practices. J Pediatr Psychol. 2007;32:960-72.
61. Larios SL, Ayala GX, Arredondo EM, Baquero B, Elder JP. Development and validation of a scale to measure Latino parenting strategies related to children's obesogenic behaviors. The Parenting Strategies for Eating and Activity Scale (PEAS). Appetite. 2009;52:166-72.
62. Wardle J., Sanderson S., Guthrie C.A., Rapoport L., Plomin R. Parental feeding style and the inter-generational transmission of obesity risk. Obesity Research. 2002; 10:453-462.
63. Ogden J, Reynolds R, Smith A. Expanding the concept of parental control: a role for overt and covert control in children's snacking behaviour? Appetite. 2006;47:100-6.
